# Cationized albumin conjugated solid lipid nanoparticles as vectors for delivery of albendazole against cystic echinococcosis

**DOI:** 10.1186/s13071-024-06473-5

**Published:** 2024-12-27

**Authors:** Fariba Faizi, Reza Mahjub, Negin Torabi, Seyedmousa Motavallihaghi, Mohammad Fallah

**Affiliations:** 1 Department of Medical Parasitology and Mycology, School of Medicine, Hamadan University of Medical Sciences, Hamadan, Iran; 2 Faculty of Pharmacy, Hamadan University of Medical Sciences, Hamadan, Iran; 3 Department of Parasitology and Mycology, School of Medicine, Zanjan University of Medical Sciences, Zanjan, Iran

**Keywords:** Albendazole, Albumin, Solid lipid nanoparticles, Hydatid cyst

## Abstract

**Background:**

Cystic echinococcosis (CE) is a common neglected parasitic disease. Nanoparticles containing drugs have been widely utilized in various formulations for several purposes, including improving the bioavailability of drugs by increasing the solubility and dissolution rate of the nanoparticles. The purpose of this study was to evaluate the effects of solid lipid nanoparticles containing albendazole and conjugated to albumin (B-SLN + ABZ) as a novel treatment approach for hydatid cysts in vivo.

**Methods:**

Albendazole-loaded solid lipid nanoparticles were prepared by emulsification and solvent evaporation method. The experimental mice were assessed for prophylactic and therapeutic effects of the drugs. Ultrastructural changes were observed by transmission electron microscopy.

**Results:**

The variance analysis of the fitted model indicated that the Glyceryl monostearate (GMS)/soy lecithin concentration ratio and the amount of albendazole had a significant effect on nanoparticle size. The GMS/soy lecithin concentration ratio and the amount of albendazole had a notable effect on nanoparticle polydispersity index (PdI) and entrapment efficiency (EE%), respectively. During chemoprophylaxis, the B-SLN + ABZ group showed a lower number and weight of cysts (0.90 ± 0.73 and 15.01 ± 10.46, respectively) compared with the ABZ + SLN group (1.4 ± 0.51 and 26.73 ± 9.92, respectively). In addition, therapeutic efficacy analysis showed a significant reduction in wet weights of metacestodes in mice treated with both B-SLN + ABZ (29.37 ± 13.82 mg) and SLN + ABZ (35.88 ± 7.49 mg) compared with the control group (59.78 ± 3.80 mg).

**Conclusions:**

The results showed that B-SLN + ABZ nanoparticles were more effective against *E. granulosus* cysts compared with free ABZ. The cysts in the animals receiving B-SLN + ABZ every 24 h showed more ultrastructural changes.

**Graphical abstract:**

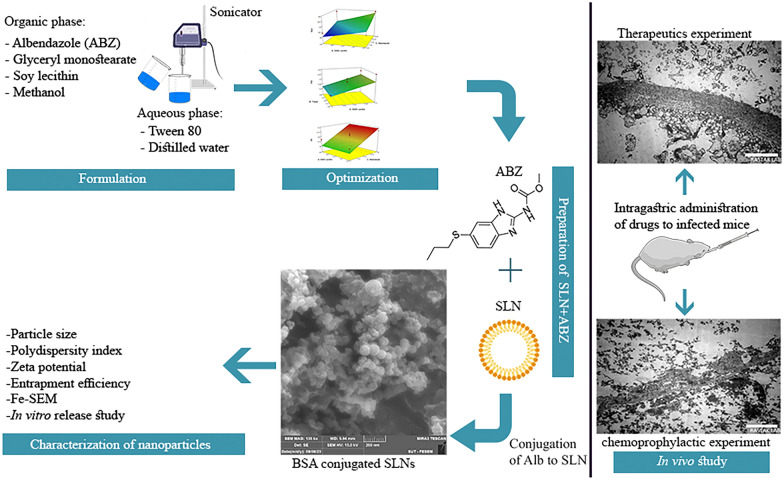

## Background

Echinococcosis is a zoonotic disease with a significant socioeconomic burden on human populations in many regions of the world and is recognized by the presence of fluid-filled cysts that primarily affect the liver and lungs. Human infection can occur through accidental ingestion of parasite eggs or direct contact with dogs. Indirect exposure may also occur through contaminated vegetables, fruits, food, soil, or water [1]. As this disease has a high prevalence and can result in various health complications, The development of a variety of diagnostic and treatment methods is essential [2]. Until the 1970s, surgery was the only treatment option for hydatid cysts. However, the discovery of benzimidazoles offered an alternative approach [3]. Chemotherapy is essential to achieve a complete cure owing to the increased likelihood of recurrence and in cases where surgery is not a viable option [4]. Benzimidazole drugs, including ABZ and MBZ, are recommended for the prevention of secondary cystic echinococcosis (CE) both before and after surgery [5]. ABZ is the preferred drug for the treatment of echinococcosis owing to its superior efficacy in chemotherapy; however, it has several disadvantages, including low solubility in water and limited absorption in the gastrointestinal tract, resulting in inadequate drug levels in plasma and hydatid cyst fluid (HCF). In addition, the limited permeability of ABZ results in inadequate drug concentrations in both plasma and cyst [6]. Reports suggest that the main reason for the effectiveness of chemotherapy in treating hydatid disease is the drug’s ability to penetrate the cyst layers and maintain a sufficient concentration within the cysts for a period of time [7]. Sophisticated nanotechnology methods have been developed to enhance the solubility and permeability of drugs, resulting in increased therapeutic efficacy and reduced side effects [8]. The membranes of hydatid cysts consist of a fibrous layer derived from the host and two layers derived from the parasite that act as barriers. The parasite layers consist of two parts: an outer laminated layer that is approximately 1 mm thick, and a thinner inner germinal layer that is 10–25 µm thick [9]. Various nanocarriers, such as micelles, liposomes, nanocapsules, and solid lipid nanoparticles (SLNs), are used to achieve controlled drug release within a specified timeframe [10]. Since the early 1990s, SLNs have been used as a drug delivery system [11]. SLNs are considered a preferred drug delivery system for lipophilic drugs due to their use of physiological lipid matrices that effectively reduce immune response and toxicity [11, 12]. Possible applications for these carriers include controlled drug release, enhanced drug stability, precise distribution targeting, improved therapeutic efficacy, and minimized unpredictable absorption [13–16]. The composition of cysts may vary depending on geographic location and the specific strains present. HCF is a mixture of host serum elements (approximately 90%) and parasite antigens [17]. It is essential for the development of parasitic larvae and plays a vital role in the life cycle of *Echinococcus* [18]. Various electrolytes, enzymes, proteins, lipids, vitamins, and hydrocarbons have been identified in HCF. The interaction between a parasite and its host is of great interest to researchers. HCF is composed of proteins secreted by the germinal layer and host proteins, particularly plasma proteins such as hemoglobin, serum albumin, immunoglobulins, and lipoproteins [19, 20]. The mechanism of transfer of host proteins to *Echinococcus* metacestode vesicles is not well understood, and the existence of endocytosis on the tegument surface of tapeworms remains an unsolved problem [21]. Even in the case of *Echinococcus*, it has been suggested that host macromolecules can enter randomly due to random leakage events from tegument degradation. Therefore, host proteins are physically capable of interacting with germinal layer cells and parasite HCF proteins [19, 20]. The presence of host proteins in HCF has been confirmed by proteomics analysis and specific antibody identification. The HCF proteins are primarily albumin and globulin of host origin. These proteins interact with the hydatid cyst [22]. Therefore, according to the evidence of the presence of host proteins in HCF and their interaction with the germinal layer and HCF, and assuming that albumin as a substance that increases the permeability of the drug into the cyst, we used it as a substance to improve the penetration of albendazole into the hydatid cyst.

## Methods

Protoscoleces were collected under aseptic conditions from naturally infected sheep liver cysts at a local abattoir in Hamadan, Iran. The viability of the Protoscoleces was evaluated by observing their movement and flame cell activity under a microscope after staining with 0.1% eosin, showing a viability range of 90–95% [23].

### In vivo chemoprophylactic experiment

Experimental infection was performed on 60 female BALB/c mice (aged 6–8 weeks, body weight 25 g ± 5) by injecting 1500 viable PSCs suspended in 0.5 ml RPMI 1640 medium into the peritoneum of each mouse using a 19-gauge needle. Mice were randomized into six equal groups of ten mice each: group 1: untreated control group receiving normal saline, group 2: SLNs loaded with ABZ and conjugated to albumin, group 3: ABZ-loaded SLNs, group 4: received SLNs without drug, group 5: free ABZ group, and group 6: albumin-treated group. The drugs were administered intragastrically (25 mg/kg) suspended in 0.3 ml normal saline. At the end of 7 months of infection, the mice were euthanized, and the cysts were promptly collected from the peritoneal cavity.

### Therapeutics efficacy study

Then, 7 months post-infection and cyst development, the mice were divided into six treatment groups as in the chemoprophylaxis study. Treatment began with intragastric administration of the drugs suspended in 0.3 ml normal saline at a dose of 25 mg/kg for 21 days. Animals were euthanized and necropsied immediately at the end of the treatment period. Hydatid cysts were carefully removed by opening the abdominal cavity. Similarly, the number, size, and weight of collected cysts were recorded as previously described [24]. Wet cyst weight was measured using an electronic balance (0.001 mg accuracy) and cyst size was measured using a caliper (1 mm accuracy). Cyst samples from each group were processed for transmission electron microscopy (TEM).

### Preparation of solid lipid nanoparticles (SLN + ABZ)

A modified method of O/W emulsification and solvent evaporation was used to prepare albendazole-containing SLNs [25]. Briefly, a mixture of soy lecithin (50 mg), albendazole (23.96 mg), and GMS (200 mg) was completely dissolved in 2.5 ml of methanol as the organic phase. The mixture was then heated to 50 °C by a Benmarry heater (Memmert VR, Schwabach, Germany). Simultaneously, the aqueous phase (5 ml) was created by dissolving 110 mg of Tween 80 as a surfactant, and then heating the solution to the same temperature as the organic phase. The organic phase was then added dropwise to the hot aqueous phase, and the mixture was homogenized under high ultrasonic waves using a probe sonicator (30 watts, pulse on/off time 30 s). The mixture was subjected to emulsification time using a magnetic stirrer (Heidolph VR, Schwabach, Germany) set at 1000 rpm to aid in the evaporation of organic solvents. The colloidal dispersion was solidified by placing it in an ice bath at 0 °C and then subjected to continuous stirring at a speed of 1000 rpm for 1 h, which was the duration of the solidification process. Eventually, an opalescent colloidal suspension was produced. The dispersion was centrifuged at 14,000 rpm for 30 min at 4 °C. The precipitated nanoparticles were assembled for later study, and the transparent supernatant was utilized to measure EE% (entrapment efficiency) and LE% (loading efficiency).

### Preparation of solid lipid nanoparticles (SLN) containing albendazole and conjugated to albumin (B-SLN + ABZ)

#### Isoelectric point

Isoelectric refers to the pH at which a molecule carries no net electrical charge (i.e., the molecule is electrically neutral overall). This point is also known as the isoelectric point (pI), where the number of positive and negative charges on the molecule are equal. The pH of the environment can affect the net charge of the molecule, causing it to increase or decrease as protons are gained or lost [26]. Proteins are composed of amino acids that have positive, negative, neutral, or polar properties, which together determine the net charge of the protein.

Proteins exhibit a positive charge when the pH is below their isoelectric point (pI), but show a negative charge when the pH is above their pI. The native structure of BSA molecules remains stable within the pH range of 4.0 to 8.0 [27]. However, when the pH falls below 4.0 or rises above 8.0, the folding conformation of bovine serum albumin (BSA) molecules undergoes a distinct change from their native structure. BSA has an isoelectric point at pH 4.5. At this pH, the surface charge is zero and BSA molecules aggregate [28]. We performed a study on the electrostatic interaction between albendazole-loaded solid lipid nanoparticles (SLNs) and BSA.

#### Phosphate buffer pH 3

A total of 3.4 g of potassium dihydrogen phosphate was dissolved in 25 ml of water and was adjusted to pH 3.0 with phosphoric acid [29]. After the synthesis of the nanoparticles and their centrifugation, 3 cc of a buffer solution were dispersed with a pH of 3 into the microtubes containing the nanoparticle sediment, mixing the contents thoroughly until a homogeneous and uniform consistency was obtained. Bovine serum albumin (1 mg/ml) was added to the microtubes containing nanoparticles with pH of 3. It was completely dissolved and incubated at room temperature for 20 min to bind the albumin. Then, it was centrifuged and the supernatant was analyzed to measure the amount of albumin by the Bradford method.

### Characterization of nanoparticles

In the case of each sample, a 1 ml volume was diluted at a ratio of 1:100 with deionized water. The sample was then analyzed. The particle size distribution, polydispersity index (PDI), and surface charges (ζ-potential) of the solid lipid nanoparticles were determined by dynamic light scattering (DLS) using a nano-zetasizer device. Measurements were performed in three replicates and the results are presented as mean ± standard deviation.

### Drug loading (DL%) and drug entrapment efficiency (EE%) measurement

After centrifugation of the SLN suspensions at 14,000 rpm for 30 min, 1 ml of the supernatant was investigated via high-performance liquid chromatography (HPLC) to assess the presence of any unbound drug (free drug).

#### HPLC method

Drug concentration was determined by HPLC method. (Ce- cil 1100 series; UK). A combination of methanol, acetic acid, acetonitrile, and water in the ratio 40:10:1:49 at a flow rate of 0.5 ml/min was used as the mobile phase. The analytical column was thermostated at 55 °C. Ultraviolet (UV) detection was performed at 286 nm. The retention time for ABZ was 18.7 min. The following equations were utilized to calculate drug loading (DL) (%) and drug entrapment efficiency (EE) (%):

1 $${\text{EE}}\,\left( \% \right) \, = {\text{ total}}\,{\text{drug}}\,{\text{content}}-{\text{free}}\,{\text{drug}}\,{\text{found}}\,{\text{in}}\,{\text{the}}\,{\text{supernatant/total}}\,{\text{drug}}\,{\text{content }} \times { 1}00$$

2 $${\text{DL}}\,\left( \% \right) \, = {\text{ total}}\,{\text{drug}}\,{\text{content}}-{\text{free}}\,{\text{drug}}\,{\text{found}}\,{\text{in}}\,{\text{the}}\,{\text{supernatant/weight}}\,{\text{of}}\,{\text{nanoparticles}} \times { 1}00$$

### Experimental design

The optimization study was conducted using Design-Expert VR software (version 7.0.0, State-Ease Inc, Minneapolis, MN) with a Box–Behnken statistical design [30] with three factors, three levels, and 15 experimental runs. The independent variables (factors) were designated as the ratio of the concentration of GMS/soy lecithin (A), Tween 80 (B), and the amount of albendazole (C). The examined responses, considered as dependent variables, were particle size (Y1), PdI (Y2), and entrapment efficiency (Y3). Table 1 presents a summary of ranges and constraints associated with both the independent and dependent variables. The selection of independent variable ranges was determined by insights from previous initial studies, while the amounts of soy lecithin, Tween 80, and albendazole were maintained at 50 mg, 110 mg, and 23.96 mg, respectively. In accordance with suggested experimental design, 15 formulations (including three centers) were created in triplicate and analyzed as presented in Table 2. The obtained data were fitted to the relevant models (i.e., linear) by one-way analysis of variance (ANOVA), the models were described using polynomial equations, and the corresponding 3D response surface plots were created by design-expertVR software. To improve predictability and minimize the number of models, a stepwise method used to eliminate parameters that were not statistically significant.

**Table 1:** Ranges and constrains of independent and dependent variables used for experimental design

Independent variables (factors)	Levels			
	−1	0	+ 1	
Numeric factors	GMS/soy lecithin (A)	0.3	2.15	4
	Tween 80 (B)	0.5	2.25	4
	Amount of albendazole (C) (mg)	5	17.5	30
Dependent variables (responses)		Constrains		
Y1 = particle size (nm)		Minimize		
Y2 = PdI		Minimize		
Y3 = drug entrapment efficiency (EE %)		maximize

**Table 2:** Suggested formulations for the Box–Behnken experimental design (*n* = 3)

Independent variables				Dependent variables		
Run	A: GMS/ soylecithin	B: Tween	C: amount of albendazole (mg/ml)	Y1: size (nm) (mean ± SD)	Y2: PdI (mean ± SD)	Y3: entrapment efficiency (EE %)
1	4	0.5	17.5	316.85 ± 53.66	0.25 ± 0.07	99.28 ± 0.11
23	2.15	4	30	417.5 ± 62.93	0.2 ± 0.01	99.42 ± 0.24
	2.15	4	5	431 ± 41.01	0.3 ± 0	99.03 ± 0.01
4	2.15	2.25	17.5	416.5 ± 71.41	0.45 ± 0.07	97.85 ± 1.04
5	4	2.25	30	386.5 ± 74.24	0.25 ± 0.07	99.38 ± 0.03
6	2.15	0.5	5	309 ± 32.52	0.25 ± 0.07	98.27 ± 0.44
7	4	2.25	5	366.5 ± 54.44	0.25 ± 0.07	97.55 ± 0.09
8	2.15	2.25	17.5	426 ± 41.01	0.4 ± 0	94.37 ± 3.00
9	2.15	2.25	17.5	388.5 ± 36.06	0.35 ± 0.07	98.14 ± 1.06
10	0.3	0.5	17.5	435.5 ± 174.65	0.4 ± 0.14	98.78 ± 0.31
11	4	4	17.5	324.5 ± 85.55	0.4 ± 0.14	97.3 ± 0.46
12	2.15	0.5	30	433.5 ± 78.48	0.35 ± 0.07	97.59 ± 0.86
13	0.3	2.25	30	460 ± 171.11	0.4 ± 0.14	97.04 ± 2.34
14	0.3	2.25	5	384.5 ± 75.66	0.45 ± 0.21	99.29 ± 0.55
15	0.3	4	17.5	329.5 ± 10.60	0.3 ± 0	99.30 ± 0.64

### Optimization and model validation

To verify the proposed fitted models and to evaluate the prediction errors, which indicate the predictability and reliability of the model, the optimized formulation recommended by the software was formulated experimentally three times (*n* = 3) and particle size, PdI, zeta potential (mV), EE, and LE were determined.

### Morphological studies

The freeze-dried nanoparticles were mounted on aluminum stubs and covered by a thin gold layer using a sputter coater for a duration of 120 s at 24 mA. Subsequently, an examination was conducted utilizing a FE-SEM (MIRA3, TESCAN Company, Berno, Czech Republic) at an increased voltage of 25 kV.

### In vitro drug release

The release test was performed using a 2.5 nm pore dialysis bag (Sigma-Aldrich) with a molecular weight cut-off in the approximate range of 12,000–14,000 daltons. The membrane was soaked in Double Distilled Water (DDW) for 12 h before use. Phosphate buffer (pH 7.4, 37 °C) was used as the release medium [31]. Lyophilized ABZ + SLN powder was dispersed in 3 mL PBS and then transferred to a dialysis bag. The two ends of the bag were then sealed and immersed in 250 mL of release medium. Afterward, it was placed in a shaker incubator adjusted to 37 °C and 150 rpm. At regular intervals (15, 30, 60, 90, 120, 180, 240, 300, 360, 720, 1440, and 2880 min), 1 ml of medium was withdrawn and immediately replaced with the same amount of PBS (pH 7.4) to maintain sink conditions. The dialysis fluid (dialysate), which was withdrawn at predetermined times, was measured with a UV spectrophotometer at a wavelength of 308 nm.

### Statistical analysis of the data

A one-way analysis of variance (ANOVA) test was conducted to compare the clinical efficacy study. Analysis was performed by SPSS software version 22 for Windows, and *P* < 0.001 was considered significant. The design-expert VR software (V.7.0.0) was used for Box–Behnken response surface design and model fitting.

## Results

### Preparation and characterization of solid lipid nanoparticles

**Fig. 1:**
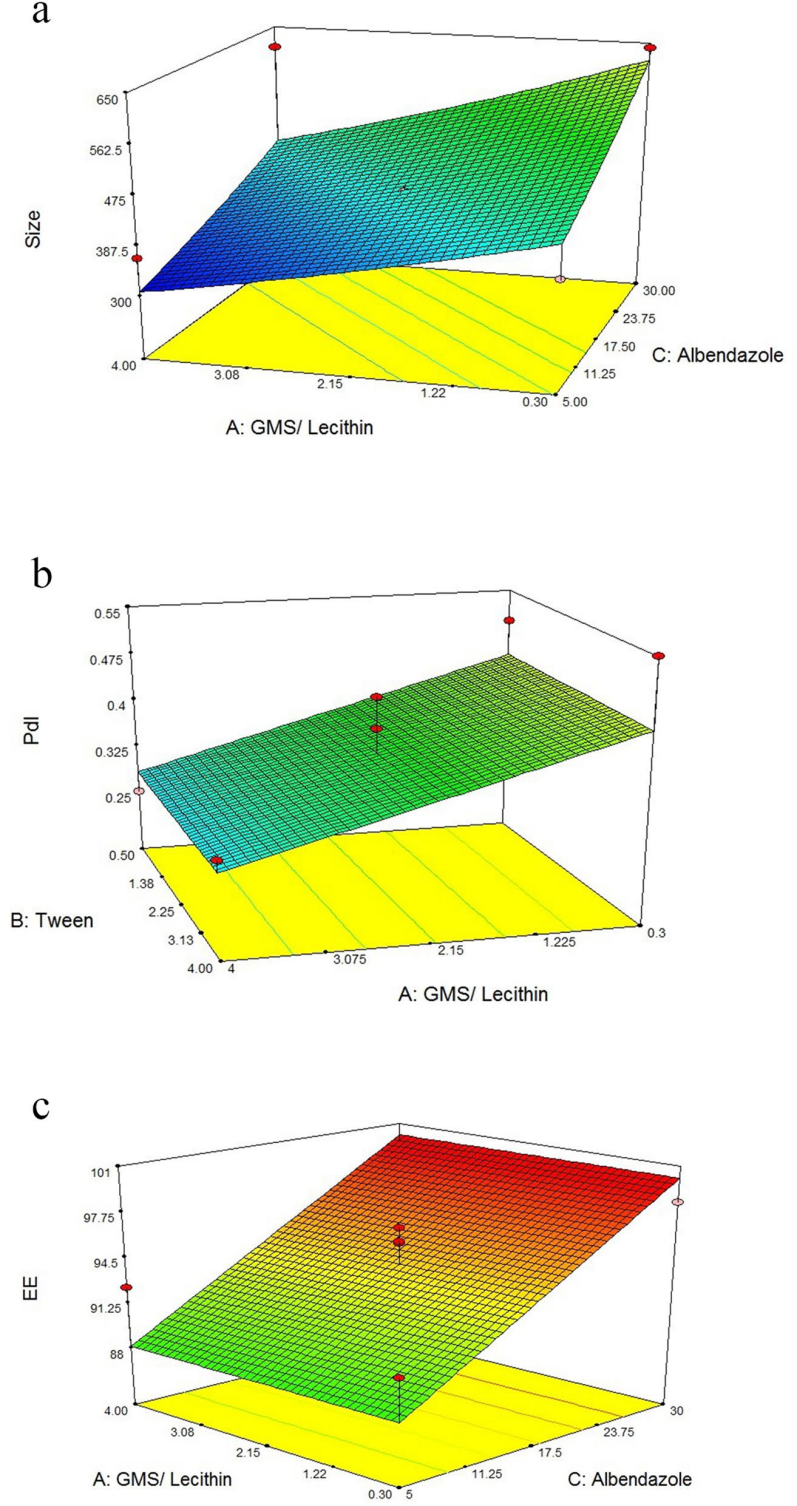
3D response surface plots of the influence of significant parameters on: particle size (**a**); polydispersity index (**b**); entrapment efficiency (EE) (**c**)

The data collected during the experimental preparation of the proposed formulations have been studied and are presented in Table 2. Polynomial equations were derived by considering the coefficients of the major factors and their binary interactions, using statistical parameters such as multiple correlation coefficients, adjusted multiple correlation coefficients, and predicted residual sums of squares obtained from the design-expertVR software. The polynomial equations were statistically validated using the software’s one-way ANOVA function. On the basis of the obtained experimental data, the optimized values of the variables were defined. To illustrate the effects of predetermined parameters such as particle size, PdI, and EE on the responses, three-dimensional (3D) response surface plots are created and depicted in Fig. 1a–c. Mathematical models were created to help explain how each factor relates to the corresponding responses.

### Particle size

The particle size varied from 309 ± 32.52 nm (formulation no. 6) to 460 ± 171.11 nm (formulation no. 13) as presented in Table 2. The ANOVA analysis was employed to examine the obtained results and determine the best significant model for predicting particle size. Table 3 indicates the properties of the linear model. The table indicated that the linear model was considered appropriate for predicting the response due to its significance (*P* < 0.05) and the lack of fit being nonsignificant (*P* > 0.05). The ANOVA results for the model indicated that two main factors, the GMS/soy lecithin concentration ratio (A) and the amount of albendazole (C), had a significant effect (*P* < 0.05) on nanoparticle size.

**Table 3:** Characteristics of the proposed models for prediction of size, PdI, and EE of particles

Dependent variables (responses)	*P*-value	Best fitted model	Lack of fit	Adeq precision	Pred *R*-squared	Adj *R*-squared	*R*-squared
Y1: particle size	0.0139	Linear	Insignificant (*P* > 0.05)	7.88	0.1416	0.4279	0.5096
Y2: PdI	0.0385	Linear	Insignificant (*P* > 0.05)	4.458	0.0235	0.2350	0.2896
Y3: EE	0.0025	Linear	Insignificant (*P* > 0.05)	7.239	0.2905	0.4810	0.5181

Coefficients of important parameters affecting particle size (Y1) are expressed in formula (3):

3 $${\text{Y1 }} = \, + {2}.{63 } - \, 0.0{43639} \times A \, + \, 0.0057749 \times C$$

Y1 is the nanoparticle size, *A* is the concentration ratio of GMS/soy lecithin as an independent variable. *B *is also an independent variable indicating the amount of Tween 80, and *C* is the amount of albendazole. Figure 1a shows a 3D response surface plot illustrating the changes in particle size in response to variations in independent variables *A* and *C*. The graph demonstrates that a higher concentration ratio of GMS/soy lecithin leads to a reduction in the mean particle diameter. Figure 1 also indicates that an increase in the amount of albendazole elevates particle size.

### Polydispersity index

As can be seen in Table 2, the PdI varied from 0.2 ± 0.01 (formulation no. 2) to 0.45 ± 0.21 (formulation no. 14). The data were evaluated using ANOVA to identify the most suitable model for predicting PdI. Table 3 summarizes the characteristics of the best fitted model.

The table presents the significance of the proposed linear model (*P* < 0.05), while the lack of fit did not reach significance (*P* > 0.05). This indicates that the suggested model is suitable for predicting the response. The variance analysis of the fitted model showed that only GMS/soy lecithin concentration ratio (A) had significant effects (*P* < 0.05) on nanoparticle PdI.

None of the remaining primary factors or binary interactions show significant effects (*P* > 0.05). The coefficients of the important variables affecting the PdI of the nanoparticles (Y2) are as described in Eq. (4):

4 $${\text{Y2}} = + 0.{45776} - 0.0{4}39 \times A$$

Here, Y2 represents the PdI of the nanoparticles, while *A* is the independent variable, the concentration ratio of GMS/soy lecithin. Figure 1b exhibits a 3D response surface plot illustrating how PdI varies with changes in *A* (as the main independent variable). The graph indicates that the PdI of the nanoparticles decreases when the concentration ratio of GMS/soy lecithin is increased from 0.3 to 4.

### Entrapment efficiency (EE)

The percentage of entrapment efficiency ranged from 99.42 ± 0.24 (formulation no. 2) to 94.37 ± 3.00 (formulation no. 8) on the basis of the experimental data presented in Table 2. The results were examined by ANOVA and utilized to suggest the most significantly fitted model for predicting EE. Table 3 presents a summary of the features of the linear fitted model. The table presents the significance of the proposed linear model (*P* < 0.05), with a non-significant lack of fit (*P* > 0.05), indicating the suitability of the proposed model to predict the response. The analysis of variance of the suggested model showed that of all the primary factors, only factor C (the amount of albendazole) had a notable effect on particle EE (*P* < 0.05). In contrast, the remaining primary factors and their corresponding binary interactions did not show significant effects (*P* > 0.05). Equation (5) illustrates the coefficients of the significant variables on particle encapsulation efficiency (Y3).

5 $${\text{Y3 }} = \, + {85}.{7}00 + 0.{48} \times C$$

Y3 is the entrapment efficiency of the nanoparticles; *C* is the independent variable of the amount of albendazole. Figure 1c depicts a 3D response surface that demonstrates the variations in entrapment efficiency in response to changes in factor *C*, which is the significant independent variable. The graph shows that as the amount of albendazole increases, so does the entrapment efficiency of the particles.

### Optimization and model validation

The physicochemical properties of SLN were optimized through statistical analysis of experimentally collected data. The optimization objective was set to minimize the particle size, reduce the PdI value, and maximize of entrapment efficiency. Table 4 presents the recommended optimized parameters for nanoparticle preparation. The optimal values for the GMS/soy lecithin concentration ratio (A), the concentration of Tween 80 (B), and the amount of albendazole (C) are predicted to be 4, 3.16, and 23.99 mg, respectively, as presented in the table. To validate the model and calculate prediction errors, the nanoparticles were synthesized and experimentally characterized in triplicate (*n* = 3). Table 4 presents the results, including observed responses and computed prediction error values. The table reveals that the computed prediction errors were less than 10%, indicating that the fitted models were meaningful, effective, and suitable for predicting responses.

**Table 4:** Optimization and model validation data

Suggested optimized independent variables			Predicated optimized responses			Experimental observed responses (*n* = 5)							
						Size (nm)		PdI		EE (%)		LE (%)	Zeta potential (mV)
GMS/soy lecithin	Tween 80	Amount of albendazole (mg)	Size (nm)	PdI	EE (%)	Observed (mean ± SD)	Error (%)	Observed	Error (%)	Observed (mean ± SD)	Error (%)	Observed (mean ± SD)	Observed (mean ± SD)
4	3.16	23.99	394.61	0.28	97.27	339.4 ± 22.94	−17.27%	0.35 ± 0.051	26.94%	99.27% ± 0.081	2.08%	15.36% ± 0.16	−26.23 ± 5.57

### Morphological studies

**Fig. 2:**
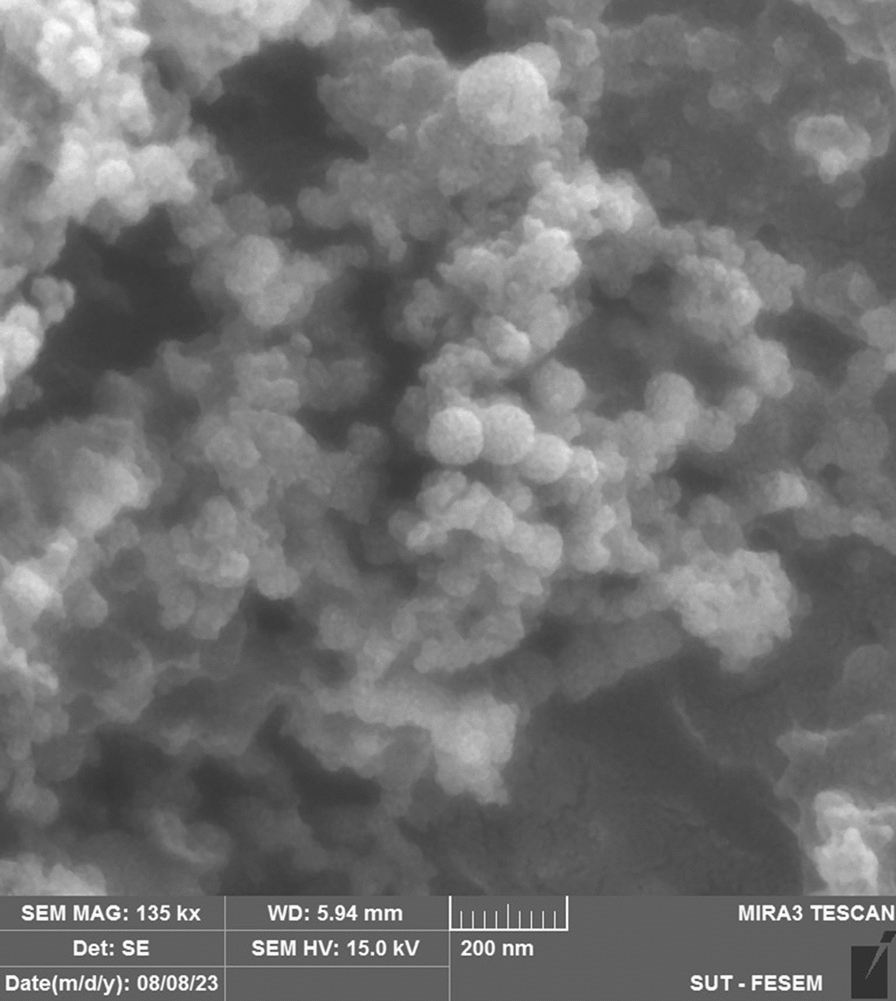
Fe-SEM images of ABZ -SLNs

The FE-SEM findings revealed that ABZ-SLN were in the nanoscale, displaying a spherical to elliptical shape with a smooth surface (Fig. 2). Moreover, the particle size determined through FE-SEM agreed well with the results gained from dynamic laser scattering (DLS).

### BSA conjugated SLNs

After five repetitions of the experiment, the percentage of albumin conjugated to SLN was 57.63%.

### In vitro release study

**Fig. 3:**
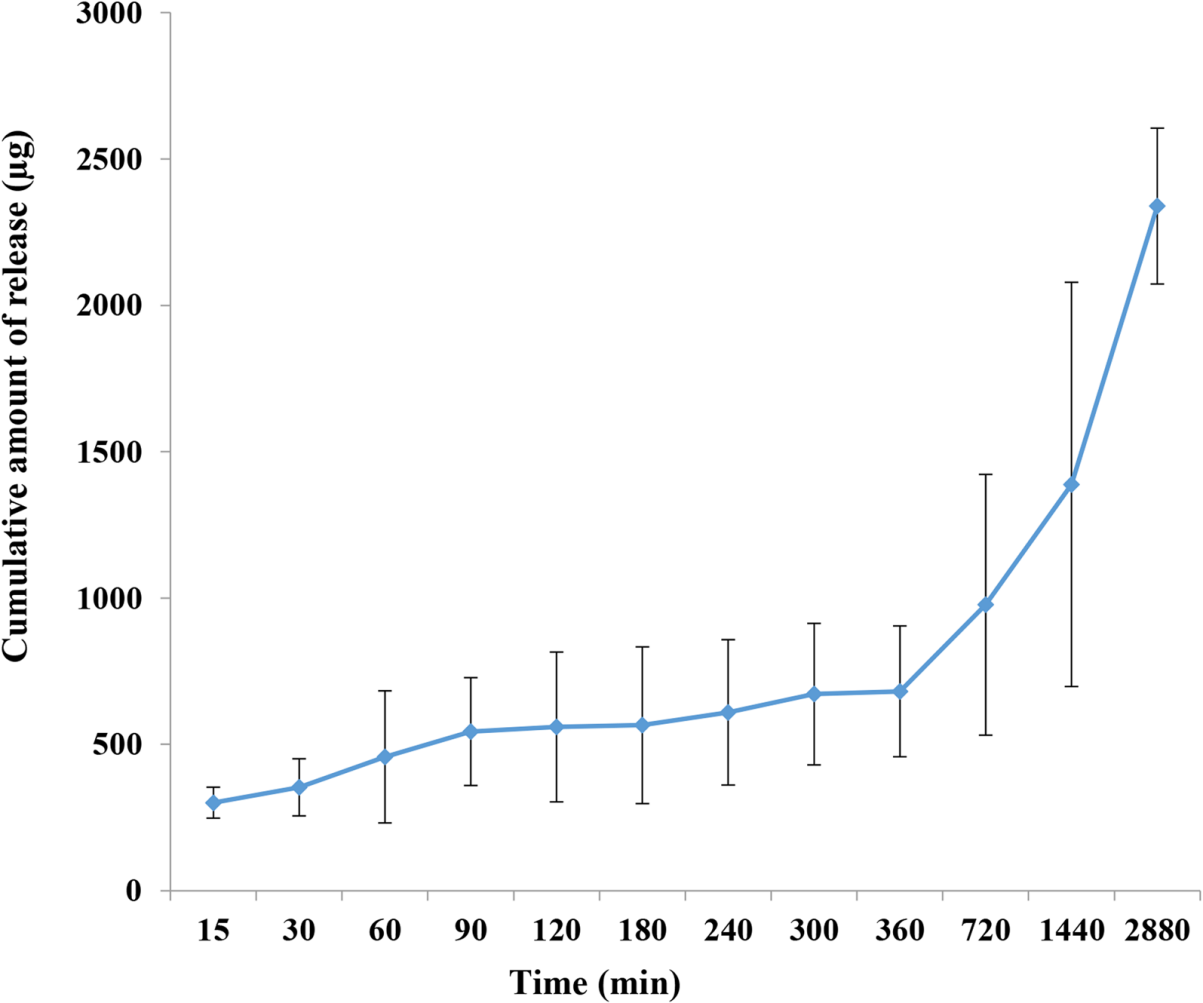
Cumulative in vitro release profile of albendazole from nanoparticles in phosphate buffer (pH 7.4) at 37 °C (*n* = 3)

The release pattern of ABZ from the optimized SLNs was determined in vitro using a dialysis membrane submerged in phosphate buffered saline (PBS) balanced to pH 7.4. The findings are shown in Fig. 3. The data showed a slight burst release (297.78 ± 20.19 µg) until 30 min post incubation. In further post-incubation time, the graph shows a sustained and prolonged release of the entrapped drug from the nanoparticles in a manner that after 360 min post-incubation, 559.52 ± 103.97 µg drug was released. Afterward, the release profile shows substantial increase and in the endpoint of the study, (i.e., 2880 min), 2416 ± 325.97 µg of albendazole was released from the prepared nanoparticles.

### In vivo efficacy study

After intraperitoneal inoculation of BALB/c mice with *E. granulosus* metacestodes and oral administration of drugs at a dose of 25 mg/kg/day, the mice were sacrificed. A thorough examination of the internal organs and peritoneal cavity was then performed to ascertain the presence of hydatid cyst infection. Cysts were collected in a Petri dish to assess their morphological changes and then compared with untreated animals from the normal control group. In simple terms, the size, number, and weight of hydatid cysts were measured and compared between the different treatment groups and the positive control group (treated with normal saline). Tables 5 and 6 present the results of therapeutic interventions and chemoprophylaxis in different experimental groups. As presented in the tables, the control group had the highest size, number, and weight of hydatid cysts in mice, whereas the mice in the B-SLN + ABZ group had the lowest size and number of hydatid cysts.

**Table 5:** Weights (mg), numbers, and sizes (mean ± SD) of hydatid cysts recovered from the infected mice of the different study groups in the chemoprophylactic treatment

Experimental group (*n* = 10, *N* = 60)	Number of cysts (mean ± SD)	Size (mm) of cysts (mean ± SD)	Cyst weight(mg) (mean ± SD)
B-SLN + ABZ	0.90 ± 0.73^a,^^b^	1.59 ± 1.13	15.01 ± 10.46^c^
SLN + ABZ	1.4 ± 0.51^a^	3.15 ± 0.53	26.73 ± 9.92
Free ABZ	3.5 ± 1.17	5.3 ± 1.14	40.10 ± 7.68
SLN	5.8 ± 1.31	8.97 ± 1.11	47.36 ± 8.00
Alb	6.3 ± 1.76	9.01 ± 1.05	54.97 ± 3.25
Untreated control	6.5 ± 1.58	9.34 ± 0.97	56.8 ± 11.73

^a^*P* < 0.001, statistically significant differences between the treated group and the control group.

^b^*P* = 0.948, statistically no significant differences between the B-SLN + ABZ group and the ABZ + SLN group.

^c^*P* = 0.053, statistically no significant differences between the B-SLN + ABZ group and the ABZ + SLN group.

**Table 6:** Weights (mg), numbers, and sizes (mean ± SD) of hydatid cysts recovered from the infected mice of different study groups in chemotherapy treatment

Experimental group (*n* = 10, *N* = 60)	Number of cysts (mean ± SD)	Size (mm) of cysts (mean ± SD)	Cyst weight (mg) (mean ± SD)
B-SLN + ABZ	2.80 ± 1.31	3.14 ± 1.42	29.37 ± 13.82^a^
SLN + ABZ	4.10 ± 0.87	4.89 ± 1.16	35.88 ± 7.49
free ABZ	4.20 ± 1.47	5.62 ± 1.77	45.52 ± 7.82^b^
SLNs	6.10 ± 1.28	7.8 ± 1.48	44.90 ± 6.88
Alb	6.60 ± 1.42	7.81 ± 1.16	54.58 ± 5.72
Untreated control	6.80 ± 1.61	8.64 ± 1.22	59.78 ± 3.80

^a^*P* = 0.49, statistically no significant differences between the B-SLN + ABZ group and the ABZ + SLN group.

^b^*P* = 0.001, statistically significant differences between the treated group and the B-SLN + ABZ group.

### Chemoprophylactic treatment

The mean number of cysts in the control group was 6.5± 1.58, while it was 0.90± 0.73 and 1.4± 0.51 in the B-SLN + ABZ and SLN + ABZ groups, respectively. This significant reduction in cyst number was statistically significant (*P* < 0.001) (Table 5). The efficacy of free ABZ in decreasing the number of cysts was 46.15%, whereas the efficacy of ABZ + SLN was 78.46%. Although the B-SLN + ABZ group had a lower number and weight of cysts (0.90 ± 0.73 and 15.01 ± 10.46, respectively) than the ABZ + SLN group (1.4 ± 0.51 and 26.73 ± 9.92, respectively, there was no significant difference between the two treated groups.

### Therapeutics efficacy

The control group had the largest hydatid cyst with a size and weight of 8.64 ± 1.22 mm and 59.78 ± 3.80 mg, respectively. ANOVA analysis revealed a significant decrease in wet weights of metacestodes in mice treated with both B-SLN + ABZ (29.37 ± 13.82 mg) and SLN + ABZ (35.88 ± 7.49 mg) compared with the no treatment group (59.78 ± 3.80 mg). While the efficacy of B-SLN + ABZ appeared superior to SLN + ABZ, parasite weight reduction was not statistically significant (*P* = 0.49). The cyst weight in the group treated with B-SLN + ABZ (29.37 ± 13.82 mg) showed a significant reduction compared with the free ABZ group (45.52 ± 7.82 mg) (*P* = 0.001).

### Ultrastructural examination by transmission *electron* microscopy

**Fig. 4:**
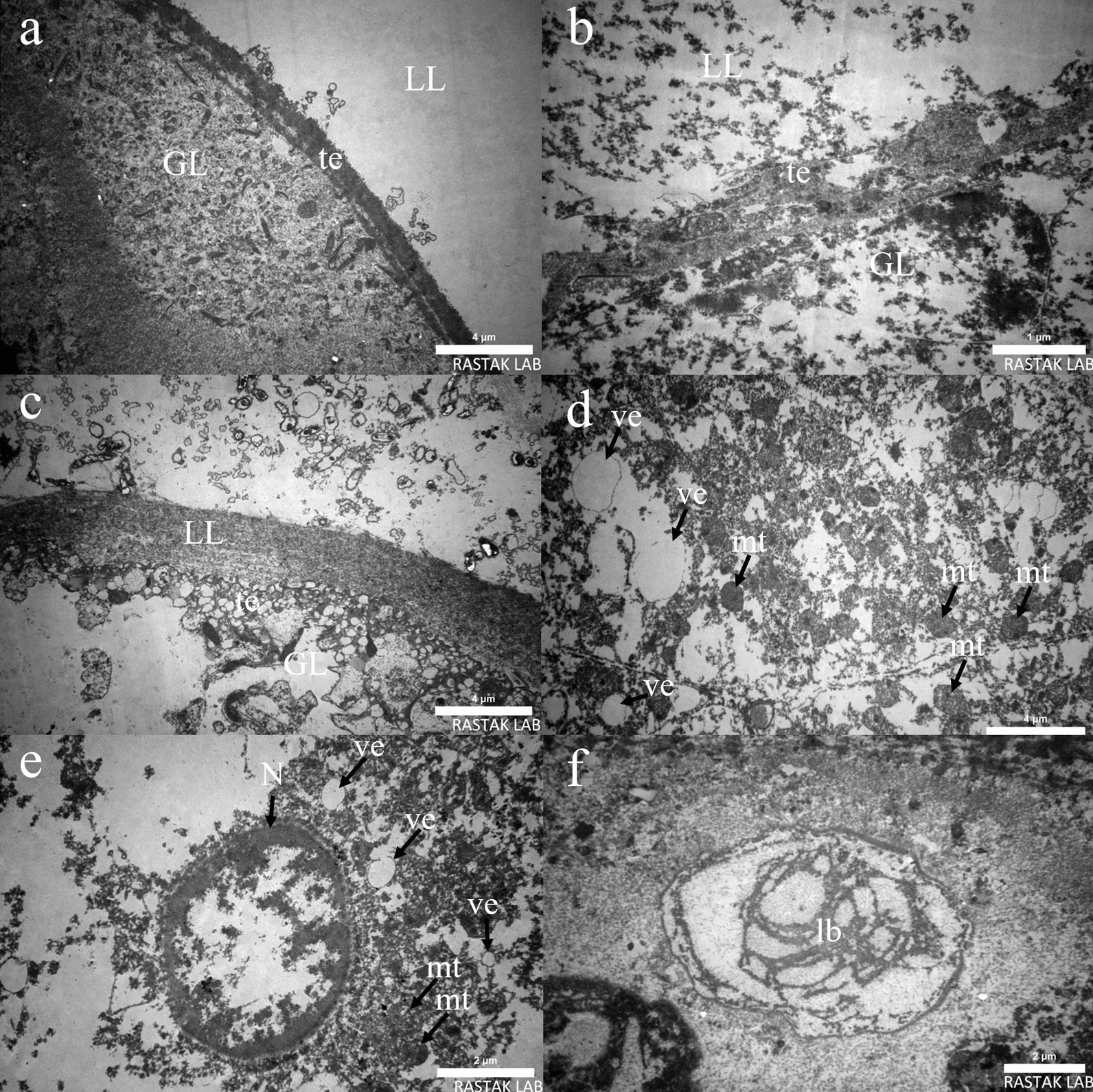
Transmission electron micrograph of hydatid cysts. **a** From an infected non treated mouse: laminated layer (LL), germinal layer (GL), and tegument (Teg) are clearly discernible. **b** Cysts recovered from B-SLN + ABZ in chemoprophylaxis group exhibited quite evident loss of microtriches; the laminated layer and germinal layer were largely destructed and only the tegumental layer is present. **c** B-SLN + ABZ in chemotherapy group; laminated layer and tegument remain, and the germinal layer has disappeared. **d**, **e** TEM micrograph of hydatid cysts from ABZ-loaded NPs-treated group showing intense destruction of the germinal layer with wide areas of vacuolated cytoplasm (ve), distorted mitochondria (mt), and nuclei (n). **f** Cysts recovered from mice treated with the free ABZ; internal tissue was affected with the presence of lamellar bodies

Transmission electron microscopy was employed to examine cysts from both treated and control mice as well as various drug formulations. TEM examination of cysts obtained from the control group showed typical characteristics of *E. granulosus* metacestodes, such as a distinct acellular outer laminated layer, intact tegument, and unaltered germinal layer (Fig. 4a). The cysts obtained from the mice treated with the B-SLN + ABZ showed marked alterations, and examination of the TEM images indicated substantial changes in the ultrastructure of the layers. In conclusion, in vivo treatment with B-SLN + ABZ for a period of 21 days induced highly similar alterations in terms of reduction in cysts weight and number as well as in ultrastructure. Parasites obtained from B-SLN + ABZ in chemoprophylaxis group exhibited distinct alterations: first, loss of microtriches was quite evident; and second, the laminated layer and germinal layer were largely destructed and only the tegumental layer was present (4b). As seen in Fig. 4c in the chemotherapy group, the laminated layer and tegument remain, and the germinal layer has disappeared. The extent of damage appears to be higher after treatment with ABZ-SLNs compared with free ABZ. ABZ-SLNs treated group in most parts of areas exhibited increased vacuolization and disappearance of microtriches and other cellular structures, as well as rounded mitochondria and decrease of complex germinal layer (Fig. 4d, e). However, treatment with free ABZ resulted in ultrastructural changes, including the presence of lamellar bodies within the inner part of the germinal layer (Fig. 4f).

## Discussion

The chemoprophylaxis study showed that treatment with B-SLN + ABZ can substantially decrease the number, wet weight, and size of hydatid cysts. The drug exhibited obvious efficacy in reducing the weight (73.61%) and number (86.15%) of cysts compared with the control group. The results showed that as the concentration ratio of GMS/soy lecithin increased, the size of the nanoparticles decreased. The observed phenomenon may be justified by considering the fact that for preparation of nanoparticles with the minimized particle size, a proper ratio of GMS/lecithin is needed. In higher or lower values of that optimized value, the particle size is increased. This study revealed that as the GMS/soy lecithin ratio increased from 0.3 to 4, the PdI decreased. Likewise, Jain et al. [32] found that a higher concentration of soy lecithin resulted in the production of SLNs with a high PdI. Under conditions of low GMS/soy lecithin ratio, an excess of soy lecithin is organized into bilayer structures, causing vesicle formation within the lipid matrix. This process leads to the production of nanoparticles characterized by elevated PdI values. However, increasing the ratio to 4 decreased PdI, as GMS molecules moved between soy lecithin structures, preventing the formation of vesicles [33]. After analyzing the experimental data using mathematical modeling and statistical prediction, the Design ExpertVR software proposed an optimized formulation that provided the smallest particle size, lowest PdI, and maximized EE%. The homogeneity of the particle size distribution in a colloidal dispersion is measured with the PdI. Narrow particle size distributions have lower PdIs, while broader distributions have higher PdIs [34]. The optimized nanoparticles have a rather homogeneous size distribution, as indicated by the experimentally calculated PdI value of 0.35 ± 0.05.This study showed that entrapment efficiency (EE %) was significantly improved by increasing the amount of albendazole. Despite numerous studies on the use of albendazole together with surgery, an effective formulation to enhance the efficacy of the drug has not yet been discovered. From this perspective, various approaches have been employed to enhance the effectiveness of chemotherapy using appropriate formulations [35, 36]. SLNs are suitable carriers and can improve solubility and target delivery. Thus, the objective of this study is to introduce the drug albendazole into nanoparticles to change the distribution pattern of the drug in the body, increase the concentration of the drug in the infected tissues, and optimize the drug treatment of hydatid cyst. Soltani et al. examined the permeability of albendazole, albendazole sulfoxide, and their SLN-loaded forms on hydatid cyst membranes. The SLN-loaded drug demonstrated superior physicochemical properties, targeted release, enhanced permeability, and greater efficacy for treating this disease compared with the other forms [37]. Another investigation evaluated the effects of a combination of albendazole and praziquantel, and their SLN-loaded form, on hydatid cysts. The findings indicated that the loaded form of these two drugs was more effective in treating the disease than their free form [38]. The findings of the current study are consistent with previous research demonstrating the efficacy of nanoparticle-loaded albendazole in treating hydatid disease in experimental animals. For instance, Ahmadnia et al. (2013) discovered that the use of albendazole sulfoxide and albendazole sulfoxide-loaded solid lipid nanoparticles (SLNs) in mice led to a decrease in cyst weight compared with control [39]. The current study showed that the treated groups (SLN + ABZ, B-SLN + ABZ) had significant reductions in hydatid cyst size, number, and weight. Among the groups, the B-SLN + ABZ group had the lowest values and the greatest efficacy rate (86.15%). In summary, the use of B-SLN + ABZ nanoparticles resulted in an antiparasitic effect against *E. granulosus* in mice during experiments. Our TEM study findings were consistent with these results, as we observed morphological changes in the cysts of the treated group that received the B-SLN + ABZ and the most significant alterations were observed in the cysts retrieved from the B-SLN + ABZ group. The efficacy of ABZ in this study was not as high as the 46.9% efficacy reported by Pensel et al. This difference could be attributed to the use of different ABZ formulations, as Pensel et al. used albendazole sulfoxide, an active metabolite of ABZ that reaches high levels within the cyst [40]. The findings demonstrated that the ABZ + SLN group had better efficacy compared with the free ABZ group. In addition, a significant decrease in cyst wet weight and size was observed in the ABZ + SLN group versus the free ABZ group (*P* < 0.05). Treatment with ABZ-loaded SLNs improved efficacy from 46.15% to 78.46% in number and from 43.14% to 66.27% in size compared with free ABZ. The current study revealed a significantly higher efficacy rate for ABZ + SLN compared with the SLN group (*P* < 0.001). Additionally, the number of developed cysts in the SLNs without drug group was significantly higher than in the SLNs loaded with ABZ group. In our study, both the number and size of cysts were reduced by 46.15% and 43.14%, respectively, with free ABZ. The timing of administration is a critical factor in determining the efficacy of albendazole on free protoscoleces when administered prior to inoculation. Albendazole is the drug most frequently prescribed for the medical treatment of echinococcosis. The findings of this study indicate that short-term (21 days) treatment with B-SLN + ABZ is effective in reducing cyst size and weight in an animal model (82.97% and 73.61%, respectively). Furthermore, SLN + ABZ was significantly more effective than albendazole alone. These findings are consistent with those of Taylor and Morris and Casado et al. [41, 42]. However, there was a difference in the timing of treatment initiation, as our experiment started 1 day before protoscolex inoculation, whereas these studies started treatment a few days after protoscolex injection. Administering drugs prior to inoculation has two advantages. First, in clinical settings, drugs are usually administered at least 1 week before surgery and continued for several months. Second, since the protoscoleces may undergo various stages of differentiation after cyst release, administering drugs before the formation of the protective laminated layer may provide a more accurate assessment of the efficacy of the protoscolicidal agent at that specific time [43]. In our animal model study, we evaluated the efficacy of SLN + ABZ in reducing cyst size and number at a low dose of 25 mg/kg and a short treatment duration of 21 days. The results showed that the drug had a significant effect, reducing cyst size by 66.27% and cyst number by 78.46% compared with the control group in the chemoprophylaxis study. In Perez-Serrano’s study, ABZ (56.9%) and ABZ sulfoxide (55.8%) had low efficacy even at a higher dose of 50 mg/kg and a longer treatment duration of 3 months. However, the combination of the two drugs resulted in a higher efficacy of 86.7% compared with each drug alone [44]. The lower efficacy observed in their study may be due to the delayed start of prophylactic treatment, which began 3 days post-infection. In contrast, administration of the same dose of ABZ 2 days prior to protoscolex injection and for a shorter duration of 1 month achieved a higher efficacy of 79.4% [45]. However, administering a higher dose of 150 mg/kg for a shorter treatment period did not result in better efficacy, as it only resulted in a 70% reduction of cysts [46]. These discrepancies in results may be due to differences in host characteristics, parasite strain, drug absorption profile and source, experimental conditions, and the various evolution stages of the parasite prior to treatment (protoscolex and hydatid cyst). The laminar layer serves as a strong barrier against drugs [47] and the physiological and immunological response of the host [48], which may also explain the results. TEM analysis showed no ultrastructural changes in the germinal layer of cysts obtained from untreated mice. However, significant changes were found in the germinal layer of cysts obtained from mice treated with B-SLN + ABZ and SLN + ABZ in both efficacy studies. It is worth noting that the administration of B-SLN + ABZ during infection (chemoprophylactic study) resulted in more extensive ultrastructural damage than when treatment was initiated 7 months after infection (clinical study). TEM analysis revealed that SLN + ABZ-treated mice exhibited significant ultrastructural changes, such as loss of the typical multicellular appearance of the germinal layer, increased vacuolation of the distal cytoplasm, and severe damage to internal tissues (Fig. 2). These findings suggest that SLN + ABZ is a more effective treatment for *E. granulosus* infection in mice than free ABZ, which may be due to the increased systemic availability of ABZ. The degenerative effect of the drugs on the parasite is reflected in the observed ultrastructural changes. ABZ inhibits the polymerization of cytoskeletal tubulins, which alters the cytoskeletal structures and affects the dynamics of vesicular traffic, ultimately leading to vacuolization of the germinal layer [49].

## Conclusions

The results of the study clearly demonstrated that a short 3-week course of B-SLN + ABZ chemoprophylaxis is more effective than albendazole alone in an animal model. This approach should be further evaluated in the clinical setting. Furthermore, the research showed that SLNs are efficient carriers of ABZ and that ABZ-loaded SLNs were more efficient than free ABZ in the chemoprophylactic treatment of cystic echinococcosis in mice. This approach could be further explored in clinical settings. It is advisable to conduct additional research to assess the efficacy of ABZ-loaded SLNs in terms of protoscolicidal effects, alternative drug delivery methods, penetration into cysts, different doses, treatment duration, and comparison with free drug.

## Data Availability

The corresponding author can provide the data upon request.

## Abbreviations

E.C: Cystic echinococcosis
ABZ: Albendazole
SLN: Solid lipid nanoparticles
SLN + ABZ: Solid lipid nanoparticles containing albendazole
B-SLN + ABZ: Solid lipid nanoparticles containing albendazole and conjugated to albumin
EE: Entrapment efficiency
PDI: Polydispersity index
HCF: Hydatid cyst fluid
TEM: Transmission electron microscopy
BSA: Bovine serum albumin
HPLC: High-performance liquid chromatography
